# An Ishmaelite

**Published:** 1888-09-01

**Authors:** Leslie Keith

**Affiliations:** Author of "The Chilcotes," "Uncle Bob's Niece," "East and West," etc.


### An Ishmaelite.

By Leslie Keith,

Author of "The Chilcotes," "Uncle Bob's Niece," "East and West," etc.

(Continued from p. 346.)

### Chapter XXI.—Taking Precautions.

When Miss Betsy had routed the enemy it is not to be supposed that she had finished her task.

She had threatened the cripple, indeed, with the direst penalties if he dared to whisper a syllable of the scandal he had ferreted out.

"My brother, John Proudie, took you out of the dirt," she said, "and I'll shut the door in your face myself if ever I hear another word of this, ye scoundrel."

Pride had a large share in Miss Betsy's sternness; she hated that even a breath of dishonour should touch the family, however remotely—the family that had always held its head so high. If the neighbours were to hear that Proudie had harboured a released convict and given him a place in his household, there would be tongues enough ready to "cast it up," make capital out of it, and gossip over it, and that contingency was more than she could bear with equanimity.

"Ye were a fool to do it, John Proudie," she said, in that interview she immediately sought with her brother. "Had ye no thought of your bairns, let alone the neighbours' tongues——"

"Preserve me," said Proudie, who was honestly distressed at the revelation, "what have the lassies got to do wi' it, woman?"

"Ye great fool!" cried Miss Betsy in accents of infinite scorn; then, seeing the cloud darken on her brother's brow, she pulled herself together. "I'll no say ye weren't bound to do Mr. Arthur a good turn since ye were beholden to him for help in your own time of need, but ye'll have to clear up this business now, John Proudie, if ye don't want that rantin' eediot Tucker to bring the street about our ears."

"Hoots!" said Proudie gruffly, annoyed and exasperated by this feminine vehemence. "Tucker must hold his tongue, and nobody 'll be a bit the worse."

"Listen to me," said Miss Betsy impressively. "Delia must go from this house this very day, an' no later. Ye owe it to Mr. Arthur and to the lassie herself to part them before there's mischeef done that's past your mending. She must go to her cousin Bruce's; she's a decent weedow woman, and will look after the bairn."

This sudden new move on his sister's part took Mr. Proudie by surprise.

He could follow the rapid workings of the feminine mind no better than the rest of his sex; a minute before she had been discoursing on his folly in sheltering a man whom no one else would have cared to harbour, and now she had flown off to Mr. Arthur and Delia. What had they got to do with the matter?

His puzzled brain refused him any clue. It takes a woman's eyes to read all those thousand little signs that tell of the inlet of love in young hearts. Affection had awakened Fred's perceptions, it is true, and revealed to him Arthur's secret; but the father—as is so often the case—was the last to dream of any danger to his child's peace. His knowledge of all the circumstances had perhaps helped to blind him. Mr. Arthur was the relative of his early employers, one of the gentry to whom Proudie gave the honest respect which he claimed for himself in his own rank. Mr. Arthur had helped him to tide over an ugly crisis in his affairs, and gratitude made him glad to serve his benefactor when chance afforded the opportunity. But he never doubted, when Arthur came to his house and took meals at his table, and was frank and friendly and genial, that he came as Fred's cousin and friend.

He never "evened" himself to Arthur, in his own phrase, nor had he any wish to bridge the social gulf that divided them. His pride would have forbidden any such attempt, and it was this pride that Miss Betsy found her best ally.

She stung him by reminding him that it would be said he had entrapped the gentleman into a marriage which his friends would resent. The censure of the world is a power even in Bermondsey. He finally gave his consent to Delia's banishment, though his heart was sore over the whole business.

Miss Betsy carried out the sentence with a Spartan-like firmness that forbade rebellion. She told Delia that it had been settled she was to go for a little to visit her cousin Bruce, who lived in a village near the sea. Poor Delia's imploring looks, her blushes, her faltering appeals had no power to reverse this verdict. Mrs. Proudie had scarcely been consulted, and the girls had not been consulted at all.

"Your father has settled it," said Aunt Betsy, and when Delia made a last appeal to him, he only said with a kind of sad awkwardness, "Yes, my little lass, you must go."

When she took her heavy heart upstairs, she found her trunk already packed. At this further proof of Miss Betsy's determination her courage forsook her. She sank down on the floor, and, laying her head on the box, sobbed out all her passionate heart.

"Oh, it is cruel, it is cruel," she said. "They might have let me be. I—I wouldn't have asked anything or expected anything——"

It was here that Barbara found her when she came upstairs before tea. As she came into the room and saw the forlorn figure on the floor her heart went out in a great pity and compassion. She knelt down, and took the little sister to her.

"Oh, Barbara," sobbed Delia, "why are they sending me away?"

Barbara could not answer. They were sending the little one out of danger, but was not the mischief done already?

"You will like being with Mrs. Bruce," said Barbara soothingly, "she is very kind, and it is near the sea. Del, you always liked the sea."

"Barbara," said Delia passionately, brushing aside this poor attempt at consolation, "you needn't talk as if it was a mere pleasure visit; you know why I am sent into exile. Oh," she broke down, "if they had let me stay I wouldn't have asked anything, only, only to see him sometimes. I know that he could never stoop to me, and that they are sending me away because they are afraid—but they needn't have been afraid——"

"They are quite right," said Barbara gently. "It might be harder for you than you think, and if it is a pain to you to go, you will bear it bravely, dear. We have all something to bear, and if this is my Del's portion, I know she will carry it with a stout heart."

Delia's hot cheek nestled against her sister's, she was silent for a time, then she looked up and said piteously:

"Bab, it isn't true what George Tucker says, that—he goes from here and—laughs——"

"No," said Barbara very sternly. "George Tucker is wicked, he has a bad heart. It is not true. Mr. Arthur Eastgate is a gentleman, he could not do such a thing. But it is just because he is a gentleman, dear, and far removed from us, that he must not come here any more. We must think of him too and of what would be for his best happiness; you would not like to—to make him unhappy, Del, or to estrange him from his friends——"

"No," said Delia. She lifted up her head and smiled bravely through her tears. "I am braver than you fancy, Barbara. I think," she said very simply, "I think he cares for me, but perhaps if I go away where he cannot see me, he will forget me, and that will be best for both of us."

"That is right," said Barbara encouragingly. "That is my good Del."

"Barbara," said the younger girl after another long pause, given perhaps to a melancholy contemplation of this heroic resolve, "George Tucker said other things about—Mr. Eastgate's cousin——"

"Yes," said Barbara, her mouth growing stern, "George has a wicked heart."

"Were they false too?"

Barbara looked straight before her with a curious light in her grey eyes. She had resolved never to betray the secret she had heard that day. She buried it deep in her heart. It was not pride that sealed her lips, it was rather a deeply-stirred tenderness.

Her absent glance presently came back and rested on Delia, "Dear," she said, "Mr. Eastgate has had a very sad life. He has had a—a great sorrow in it that sets him apart and makes him different from other men. You must not ask me any more, Delia; you must not try to think of what this—calamity is. You must not ask anyone else, or seek to find out. These things are not for us to meddle with, or-- to judge," she added in a lower voice. "It is enough for us to remember that he has been very unhappy."

"I will ask no questions," said Delia simply, "except just this one. Is he going away?"

"I think not, father will wish him to remain."

"It is George Tucker who should be sent away."

"He will not transgress again," said Barbara severely. "He knows that he must leave us if he does. Your going away will relieve you of his persecutions, Delia."

"Yes," said Delia with a forlorn smile, "I am running away from George Tucker, whom I hate, and—from—Oh, Bab——" she hid her face once more on her sister's shoulder to conceal the tears that would spring. "I mean to be good and —and brave as you are."

Perhaps that far-off look she had surprised on her sister's face had told her that there might be something too for Barbara to bear, some portion of the sadness that falls to every lot.

She did not say any more, and she tried to look cheerful when the final moment of parting came and her exile began. She had not so much as a word or a look for George Tucker, who was wild at the thought of losing her. She passed him by with a disdainful head, but it went hard with her resolve to be courageous when it came to the other farewells.

Miss Betsy cut them short with a relentless hand.

"The cab is waiting, Delia," she said; "and, let alone the poor beast standing in the cold, the man will charge extra if you're not quick."

The little trunk was secured already beside the driver, Miss Betsy's poke bonnet environed her resolute face; the Miss Greens ceased their piano-pounding to lean out of the window and stare at this exit, and the cripple stood on the door-step devoured with pangs of rage and sorrow.

So all those worthy people conspired to crush this poor little heart, and repress its yearnings and curb its natural longings, because of duty, forsooth, and because of the world's opinion—the opinion of Bermondsey and of Belgravia, which join issue, and are at one on such a count as this.

### Chapter XXII.—Disillusioned.

Arthur knew nothing of these things, because he had been bidden to Ella's marriage, which took place down in Norfolk. Cousin Fanny had written him a very urgent letter, requesting him to be present.

"Surely you won't desert us," she wrote. "You have a right, I know, to feel aggrieved, and indeed, Ella's conduct has sadly vexed both her father and me. But you have nobly forgiven her, dear Arthur, and I hope you will still further prove it by paying us a little visit and remaining after the wedding."

Arthur laughed when he read this letter. Perhaps he thought it a little too kind, but he wrote back, and said that he would not fail to witness the ceremony. He presented Ella with the handsomest bracelet he could find in Bond Street, and felt, indeed, no grudge at all against her, such as Cousin Fanny seemed to think he might harbour.

"We must console him," Cousin Fanny was saying, down at the Rectory. "We must get him a wife, since Ella has forsaken him."

Clara, who was present when this remark was made, blushed up a painful red. Poor girl! Perhaps she was tired of being offered to gentlemen who scarcely seemed to appreciate her charms.

"If mamma thinks I am going to be—kind to Arthur, she is quite mistaken," she said to her next sister and confidant, in the privacy of their own room, daring to rebel now that she was out of mamma's presence.

"Then it's a pity you got a new dress," said Fanny, junior, who was a very matter-of-fact young person.

Arthur was kind and good-humoured to all the cousins, and brought them pretty gifts from town. Indeed, he had it in his heart to be good to all the world at this juncture, but he speedily disabused Cousin Fanny of the belief that he required consolation.

"Ella's conduct was indefensible," she said, walking Arthur round the garden on the night before the wedding. "It was you whom we hoped to welcome as a bridegroom, dear Arthur, and though Ella is my child, all my sympathies are with you."

"Indeed you mustn't pity me," said Arthur, turning to her with a smile. "Ella and I quite understood each other, and she has chosen very wisely. Penfold will make her the best of husbands."

"You bear your disappointment very nobly, Arthur," said Mrs. Coates, looking hard at him, "but you cannot save Ella's father and me from self-reproach ——"

Arthur's laughter broke out at this picture of himself as an inconsolable lover cherishing a secret wound.

"Do I look heartbroken?" he said, turning a bright face towards her. "I forgave Ella long ago, if there was anything to forgive, and—she has forgiven me. We are the best of friends."

"Arthur," said Mrs. Coates with an indescribable edge of anxiety in her voice, "is there—is there anyone else?"

He understood her and was very sorry for her, and a little embarrassed and ashamed to have taken this unpermitted peep into the workings of her mind. He knew nothing, of course, of the devices that had been spent on poor Clara's toilet, but he somehow felt that he was helping to pull down another palace of Cousin Fanny's building, and his conscience smote him guiltily.

He glanced straight before him and avoided looking into his companion's sharp, eager face.

"There is someone in London, whom I hope to win some day," he said.

There was a pause, during which he seemed to hear the fall of the masonry as this Spanish castle too tumbled about the builder's ears; then she said in a cold thin voice—

"Oh, in London? Well I hope you will choose wisely, someone who will be a credit to the family."

"I wish I hadn't spent so much on Clara's gown," she was thinking, and then she reviewed the coming guests and wondered if there was anyone else with whom it would be better to pair Clara at the breakfast. Arthur could take one of the younger girls.

While these calculations were going on, Arthur's thoughts too had gone off upon a journey.

"Someone who will be a credit to the family," he was repeating, and he smiled as he thought of Delia. The world would call his choice a blunder, if nothing more severe was said of it; probably it would say that he had outraged every feeling of society. Well, he could bear its condemnation.

As he walked in the green garden he thought of her as he had thought of her in London streets too, and indeed everywhere and always. He had forgotten poor Fanny, and her new defeat, and was back in London in the humble home where all his hopes and desires were centered. He did not know that there too the world's opinion was law, and that its cruel decrees had spirited away his little Delia where he could not find her.

Nellie arrived early next morning, to be present at the wedding.

Her husband was not with her; Nellie scarcely made any excuse for his absence, though Cousin Fanny was quite shocked that she should travel alone.

"It looks so odd," she remarked severely. "And I really think, out of respect to us Mr. Bellynge might have come."

"Yes," said Nellie, showing no offence, "I think so too, but Richard never cared much for any of my relations."

A curious change had come over Nellie, and Arthur was uncomfortably struck by it. She had been from home on a round of visits, and he had seen little of her for a month or two. She looked ill, and as if she had bad nights and slept brokenly.

"She has fallen off dreadfully," said Cousin Fanny. "All that gadding about and late hours are ruinous to the constitution; I wonder her husband allows it."

Arthur had no chance to talk to his sister till the ceremony which converted Ella into Mrs. Penfold was over, and the pair had set out on their honeymoon bliss.

Nellie looked so ill that Arthur insisted she should accept Cousin Fanny's hospitality for the night, and he walked himself to the village and telegraphed to his brother-in-law.

"I will take you home myself to-morrow," he said; for his visit was to terminate then, and Cousin Fanny did not now press him to prolong it. In the morning, when the girls were busy restoring the chaos of the vicarage to order, folding away finery and counting up the spoils from the feast, he drew Nellie into the garden.

The little church where yesterday's solemnity had taken place was but a stone's throw off, and a path at the end of the garden led to the churchyard that skirted it.

Arthur guided Nellie's steps there, where the peace and quiet promised freedom from interruption.

"What is it, Nellie?" he asked.

She turned her dark eyes on him listlessly, but she made no answer.

"You are ill, Nellie; you are changed."

"Oh, no, I am not ill," she said, "I am quite well."

"Richard ought not to have allowed you to come."

"Richard doesn't care where I go,' said Nellie, a hint of stirred feeling trembling in her voice. "He is very angry with me."

"Angry with you!" echoed Arthur. "What for?"

He had come out with a half-formed intention of confiding in Nellie, of securing her sympathy, but he put this resolve aside now. It was Nellie, seemingly, who required sympathy, and he would not fail her.

He refrained from uncomplimentary remarks about his brother-in-law, though the repression cost him something of an effort.

"What have you done to displease him?" he asked.

Nellie drew a step nearer: her face was stirred with emotion now, her lips were tremulous, and her eyes had a strange light in them.

"It was about Fred," she whispered. "He found out—he questioned Briggs; he found out that I went down to Bermondsey."

"Well?" questioned Arthur fiercely.

But Nellie broke down, the long effort to appear composed and indifferent had been too much for her. She hid her face in her hands. "Arthur," she cried, "why did you urge me to go to him? He is changed, he is wholly different, he has forgotten me. Oh, I am very miserable!"

Arthur looked at her dumbly, a cold creeping doubt at his heart. Was Nellie—did Nellie—he could not frame his thoughts in any words, but he felt that this poor timid, clinging Nellie had reached a crisis in her history, and was hovering on the verge of a tragedy. He could not speak for a moment, but presently all his forces went out in a rushing desire to save her while yet she wandered bewildered on the brink.

"Changed! yes," he said. "An experience such as his would change most men; it was another world Fred woke up to when he came back to us. Haven't we changed too? Fred knew that you had married, Nellie, and the news was a comfort to him."

"A—comfort?" She let her hands fall while she looked at him.

"Yes, and why not? He was glad to know that you were happy, that you had found someone else to love. But you shouldn't have gone without Bellynge's knowledge, Nellie."

"Richard never would have let me go," she said, looking with forlorn reproach at her brother. "And—you wished it, Arthur."

"I wished you to do it openly," said Arthur, using a sternness he did not feel. "You don't suppose it could be very pleasant for Fred to feel that he had caused a coldness or a division between you and Bellynge."

Poor Nellie! she was smarting under a dreadful sense of humiliation: she looked at Arthur with a kind of tearless dismay and shrinking as from a new and bewildering pain.

"Listen, Nellie," he said, feeling that to be kind he must be cruel. "You could not wait for Fred—that was out of the question, and you had every right to choose for yourself; but you will be as glad to know as I am that some day—I hope and pray that I am not wrong—that some day there will be someone who will be to Fred what you and I could not be—a good and brave woman who will restore him to hope and a place in the world, where he may begin again." He paused abruptly.

She kept her eyes fixed on him with that strange, frightened look—all her illusions collapsed at a stroke. Her white lips could not utter a word.

Arthur felt a great pang of pity as he looked at her.

"Come, Nellie," he said putting an arm round her, "come, and I will take you home to Richard and the boy."

*(To be continued.)*

